# Clinical and radiographic outcomes of stand-alone oblique lateral interbody fusion in the treatment of adult degenerative scoliosis: a retrospective observational study

**DOI:** 10.1186/s12891-022-06035-9

**Published:** 2022-12-27

**Authors:** Yu Zhang, Chen Liu, Xin Ge

**Affiliations:** 1 School of Medicine, Shandong University, No. 44 Cultural West Road, Lixia District, Jinan City, 250012 Shandong Province China; 2 Anhui Provincial Hospital, Shandong University, Hefei, 230001 Anhui China; 3 Spine Research Center of Wannan Medical College, No.22 Wenchang West Road, Wuhu, 241001 Anhui China; 4 Department of Spine Surgery, First Affiliated Hospital of Wannan Medical College, No. 2 Zheshan West Road, Wuhu, 241001 Anhui China; 5 Department of Spine Surgery, Anqing first people’s Hospital, No. 187 Huazhong Road, Anqing, 241001 Anhui China

**Keywords:** Stand-alone OLIF, ADS, VAS, ODI

## Abstract

**Background:**

Open fusion and posterior instrumentation has traditionally been the treatment for adult degenerative scoliosis (ADS). However, minimally invasive treatment such as oblique lateral interbody fusion (OLIF) technique was developed as a new therapeutic method for the treatment of ADS. In addition, it is associated with decreased blood loss and shorter operative time without posterior instrument. The purpose of this study was to evaluate the efficiency of stand-alone OLIF for the treatment of ADS in terms of clinical and radiological results.

**Methods:**

A total of 30 patients diagnosed with ADS who underwent stand-alone OLIF in our hospital from July 2017 to September 2018 were enrolled in the study. Scores from the Visual Analogue Scale (VAS) and Oswestry Disability Index (ODI) obtained preoperatively and at the final follow-up were compared. Radiography and computed tomography were performed preoperatively and at the final follow-up. The coronal cobb angle, lumbar lordosis, disc height, sacral slope, pelvic incidence and Pelvic tilt were recorded at each time point.

**Results:**

The study cohort comprised 30 patients with a mean age of 64.5 ± 10.8 years and mean follow-up of 19.3 ± 4.2 months. The mean operative time was 96.8 ± 29.4 minutes and the mean estimated blood loss volume was 48.7 ± 9.4 ml. The mean coronal Cobb angle was corrected from 15.0° ± 3.7° preoperatively to 7.2° ± 3.1° postoperatively and 7.2° ± 3.3° at final follow-up (*P* < 0.0001). Lumbar lordosis significantly improved from 32.2° ± 11.3° preoperatively to 40.3° ± 11.8° postoperatively and 40.7° ± 11.0° at final follow-up (*P* < 0.01). The respective mean sacral slope and pelvic tilt improved from 26.1° ± 8.1° and 25.1° ± 6.9° preoperatively to 34.3° ± 7.4° and 19.2° ± 5.7° at final follow-up (*P* < 0.001). The mean disc height (defined as the mean of the anterior and posterior intervertebral disc heights) increased from 0.7 ± 0.3 cm preoperatively to 1.1 ± 0.2 cm at final follow-up (*P* < 0.0001). The interbody fusion rate on CT was 93.3%. The mean VAS pain score improved from 5.3 ± 0.6 before surgery to 2.3 ± 0.6 at final follow-up (*P* < 0.001). The mean ODI improved from 29.9% ± 6.8% preoperatively to 12.8% ± 2.4% at final follow-up (*P* < 0.001).

**Conclusions:**

Stand-alone OLIF is an effective and safe option for treating ADS in carefully selected patients.

**Trial registration:**

The study was registered in the Chinese Clinical Trial Registry (ChiCTR2100052419).

**Supplementary Information:**

The online version contains supplementary material available at 10.1186/s12891-022-06035-9.

## Background

Adult degenerative scoliosis (ADS) is a common clinical disease that is characterized by a Cobb angle of more than 10° in the coronal plane and affects approximately 6% of people over the age of 50 years [1]. ADS is caused by asymmetrical degeneration and hyperplasia of the facet joints, leading to pathological changes such as spinal instability and deformity [2]. Surgery may be required to manage intractable pain, neurological deficits, and progressive deformity [3, 4]. At present, the surgical treatment options for ADS include osteotomies, posterior lumbar interbody fusion (PLIF), transforaminal lumbar interbody fusion (TLIF), and anterior lumbar interbody fusion (ALIF). Among these surgeries, PLIF and TLIF have more neurological complications and involve paraspinal muscle stripping and dural adhesion, which may seriously affect postoperative outcomes [5]. The ALIF process may increase the risks of vascular injury and injury to the superior hypogastric plexus near the aortic bifurcation, which may lead to excessive blood loss and retrograde ejaculation [6, 7]. During the last decade, extreme/direct lateral interbody fusion (DLIF/XLIF) has attracted increasing attention as a method for correcting deformity in patients with ADS. DLIF/XLIF effectively restores coronal balance [8, 9]. However, DLIF/XLIF requires the surgeon to access the intervertebral disc via a lateral approach through the psoas major; this damages the psoas muscle and lumbar plexus, resulting in postoperative pain and paresthesia of the lower limbs [10].

In 2012, Silvestre et al. [11] reported oblique lumbar interbody fusion (OLIF) through the anterior peritoneal approach between the anterior aspect of the psoas muscle and the iliac vessels, thus avoiding direct damage to the psoas major and lumbar plexus. OLIF combined with supplemental instrumentation has been used to treat ADS. Beng et al. [12] reported that interbody distraction by OLIF with cortical bone trajectory screw fixation corrected the misalignment in 28 patients with adult spinal deformity [13]. However, in comparison with OLIF with screw fixation, stand-alone OLIF without posterior instrumentation has a shorter operative time and decreased blood loss volume, which aid in recovery. In addition, Agarwal et al. [14] showed that stand-alone lateral lumbar interbody fusion was safe and effective in 55 patients with a 10-year follow-up, while Malham et al. [15] suggested a clinical pathway to select suitable patients for stand-alone XLIF. The aim of the present study was to evaluate the clinical outcomes and radiographic correction of coronal deformity and lumbar lordosis (LL) after stand-alone OLIF in patients with ADS.

## Methods

### Study design and patient population

**Fig. 1:**
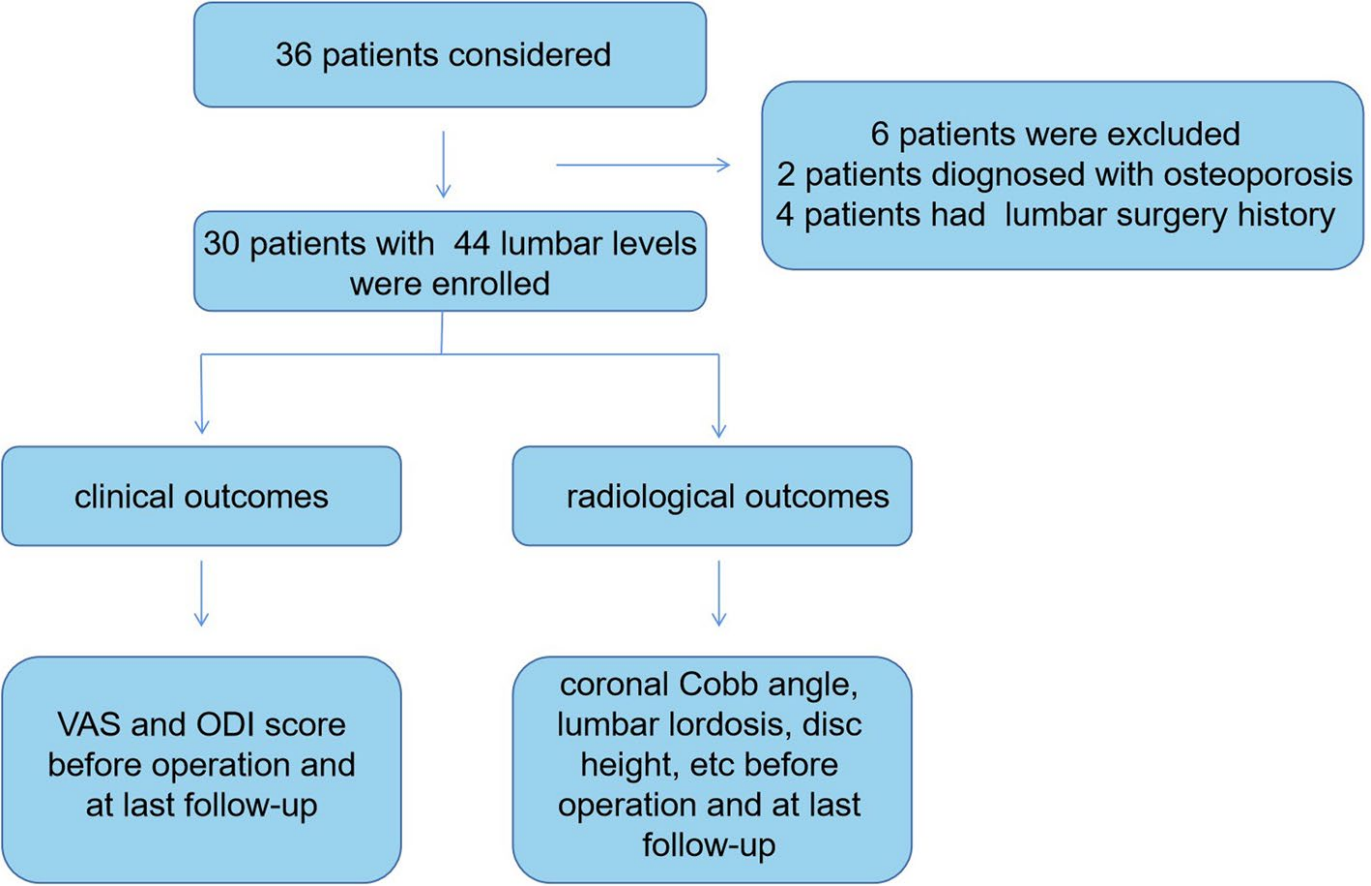
Study design of the study

The study was designed to evaluate the efficacy of stand-alone OLIF for the treatment of ADS by comparing the preoperative clinical and radiological outcomes with the outcomes at final follow-up. The clinical trial registration number was ChiCTR2100052419 (The registration time was 25/10/2021). The clinical outcomes included the visual analogue scale (VAS) pain score and the ODI; the radiological outcomes were the coronal Cobb angle, LL, disc height (DH), sacral slope (SS), pelvic incidence (PI), pelvic tilt (PI), and interbody fusion rate. The description was showed as Fig. 1. This research was approved by the IRB of the authors’ institution.

We retrospectively reviewed the medical records of all patients with ADS who underwent stand-alone OLIF from July 2017 to December 2019, and summarized and analyzed their clinical and radiographic outcomes. Inclusion criteria: (1) degenerative scoliosis mainly located in L1–L5; (2) Lenke-Silva grade I–V and no need for posterior osteotomy surgery [16]; (3) chronic lower back pain with or without lower limb radiative pain that was unresponsive to conservative treatments performed for at least 6 months. Exclusion criteria: (1) osteoporosis (bone density T value < − 2.5); (2) traumatic kyphosis; (3) inflammation and tumor; (4) previous lumbar surgery. A final total of 30 patients (17 women and 13 men) met the eligibility criteria and were included.

### Surgical procedure

The procedure was performed under general anesthesia with no intraoperative neuromonitoring. A transverse skin incision of 4 cm was made in the left lateral abdomen in the same horizontal plane as the target intervertebral disc. For multilevel cases, the incision was centered between the surgical levels. The abdominal wall muscles were bluntly separated. The retroperitoneum was entered by blunt separation with the fingers; the psoas was then retracted posteriorly, and the abdominal vessels were retracted anteriorly. A guidewire was inserted in the middle of the target intervertebral disc under C-arm guidance. Sequential dilators were placed over the guidewire, then a lighted retractor was placed over the dilators and fixed to the vertebral body with a pin to expose the operative field. The annulus fibrosus and nucleus pulposus were resected. The cartilage endplates were then curetted and rasped to expose the bony endplates. A wide and lordotic intervertebral fusion cage (Medtronic Clydesdale, Memphis, Tennessee) packed with allograft bone was inserted into the target disc under C-arm guidance. The wound was closed in layers.

### Assessment of clinical and radiographic outcomes

**Fig. 2:**
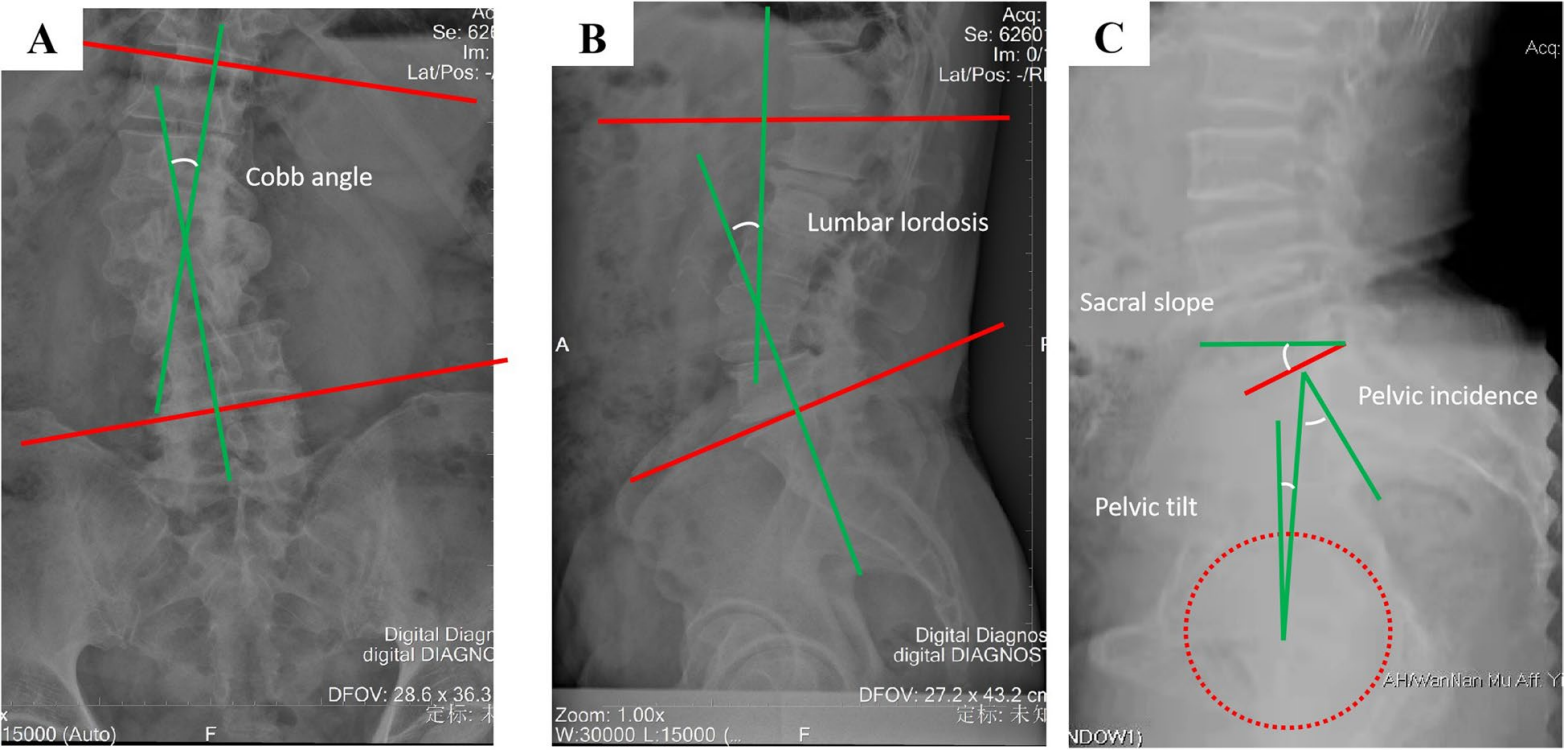
Measuring method of Cobb angle (**A**), Lumbar lordosis (**B**) and Sacral slope, Pelvic tilt and Pelvic incidence (**C**)

The operative time, intraoperative blood loss volume, hospital stay, follow-up duration, and complications were recorded. The VAS pain score and ODI before the operation, after the operation, and at final follow-up were compared to evaluate the clinical efficacy. The radiographical outcomes were evaluated on neutral anterior-posterior, lateral thoracolumbar, and full-length spinal radiographs. The scoliosis Cobb angle, PT, PI, LL, SS, and DH were measured on full-length spinal radiographs taken before and after the operation and at final follow-up. The DH was calculated as the mean value of the leading and trailing edge heights of the intervertebral disc. Lumbar interbody fusion was assessed on thin sagittal and coronal CT slices obtained 1 year after surgery. The specific measurement method is shown in Fig. 2. The criteria used to evaluate the subsidence of the cage were the same as those described by Abdala et al. [17] The degree of cage subsidence was classified based on loss of postoperative DH on a lateral radiograph of the lumbar spine as grade 0 (0–24% loss of postoperative DH), grade I (25–49%), grade II (50–74%), and grade III (75–100%). All measurements were performed by independent radiologists using a PACS system.

### Statistical analysis

Statistical analysis was performed using SPSS19.0 software (IBM Corp., Armonk, NY). All data were expressed as mean ± standard deviation. Paired t-tests were performed to compare the outcomes. In all analyses, *P* < 0.05 was considered to indicate statistical significance.

## Results

### Patient demographics, operative data, and complications

Thirty patients (17 women, 52.4%) with a mean age of 64.5 ± 10.8 years were enrolled in the study. A total of 44 lumbar levels were treated, including 16 single-level and 14 double-level cases. The mean operative time was 96.8 ± 29.4 minutes, mean estimated intraoperative blood loss volume was 48.7 ± 9.4 ml, mean hospital stay was 9.4 ± 2.9 days, and mean follow-up duration was 19.3 ± 4.2 months.

One patient (1/30, 3.3%) experienced an intraoperative vascular injury. The bleeding point was quickly found and pressed with two periosteal strippers. After hemostasis was achieved by bipolar electrocoagulation, the titanium frame was ligated and no postoperative massive bleeding occurred. Two patients (2/30, 6.7%) had postoperative pain and numbness in the anterior aspect of the left thigh; the symptoms were relieved after 1 week of treatment with drugs. Five patients (5/30, 16.7%) were found to have cage subsidence, which was the most common complication of stand-alone OLIF. The cage subsidence was regarded as grade 0 in all five patients, and no patient required second-stage posterior pedicle screw internal fixation and decompression. All five patients were successfully treated with conservative therapy during follow-up. The demographic characteristics and complications are presented in Table 1.

**Table 1:** Patient demographic, treatment information and complications

Characteristic	Statistic (***n*** = 30)
Mean age (year)	64.5 ± 10.8
**Female/Male**	17/13
**Levels per operation**	
One level (% of cases)	16 (53.3)
Two levels (% of cases)	14 (46.7)
**Levels treated**	
L2/3 (% of levels)	2 (4.5)
L3/4 (% of levels)	12 (27.3)
L4/5 (% of levels)	30 (68.2)
**Duration of surgery (range)**	96.8 ± 29.4 (50-180) (minutes)
**Blood loss (range)**	48.7 ± 9.4 ml (20-80) (ml)
**Hospitalization**	9.4 ± 2.9 (6-19) (day)
**Follow-up (range)**	19.3 ± 4.2 (12-26) (month)
**Complications**	8
Left thigh anterolateral pain (% of cases)	2 (6.7)
Vascular injury (% of cases)	1 (3.3)
Cage subsidence (% of cases)	5 (16.7)

### Clinical outcomes

The mean VAS pain score (2.3 ± 0.6 points) and ODI (12.8 ± 2.4%) at final follow-up were both significantly lower than the preoperative values (VAS score: 5.3 ± 0.6 points; ODI: 29.9 ± 6.8%) (*P* < 0.001) (Table 2).

**Table 2:** VAS score and ODI index

Before operation		Last follow-up	*P* value
VAS	5.3 ± 0.6	2.3 ± 0.6	<0.001
ODI	29.9% ± 6.8%	12.8% ± 2.4%	<0.001

### Radiographic outcomes

**Fig. 3:**
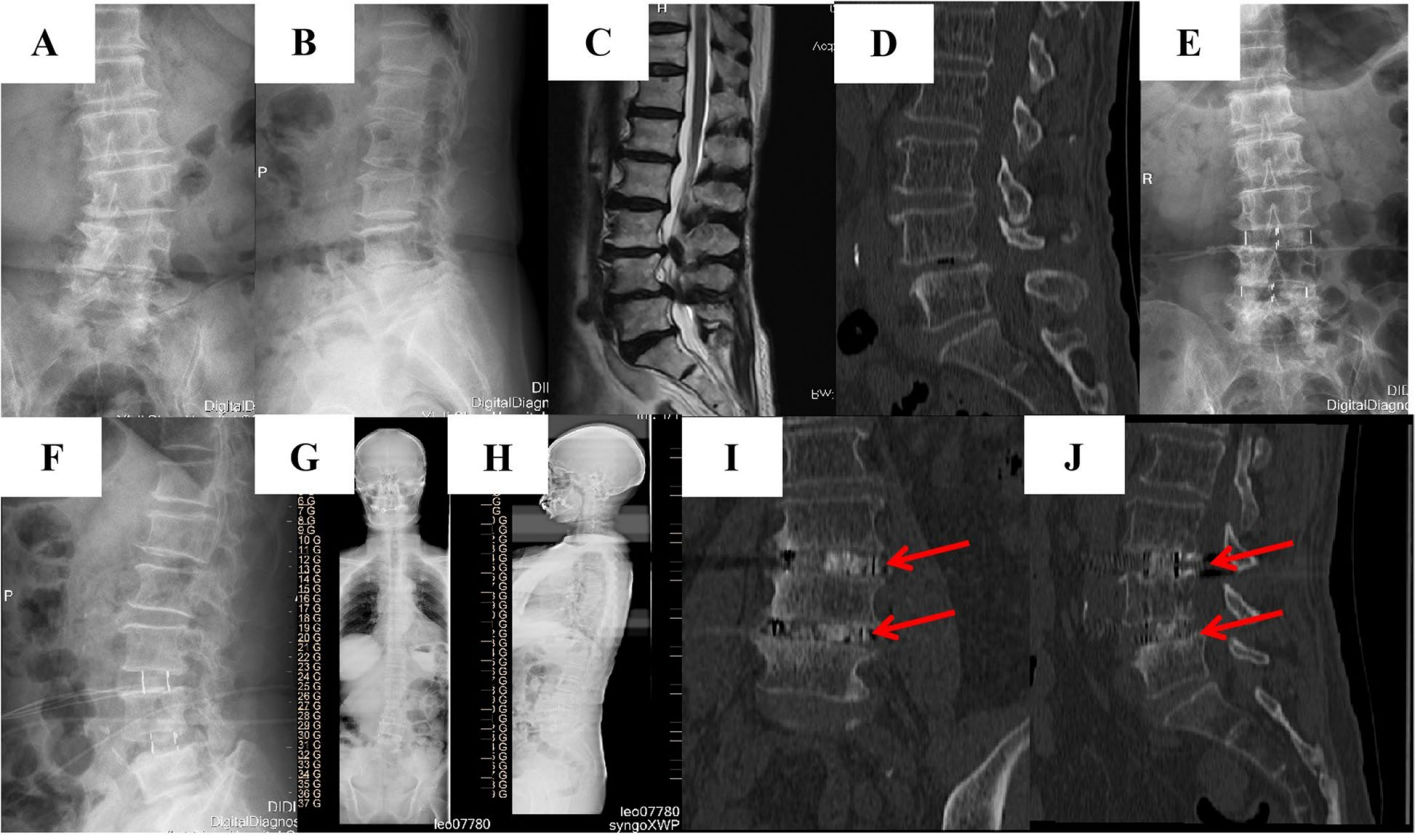
A typical case of a 66-year-old patient presented with mechanical back pain. The preoperative radiograph indicated degenerative scoliosis (**A**-**B**), preoperative MRI and CT showed lumbar stenosis of L3/4 and L4/5 (**C**-**D**). Postoperative X-ray (**E**-**F**) and standing Full length film of spine (**G**-**H**) after 1 year showed that the coronal and sagittal balance had been corrected. The 3D-CT scan indicated bony fusion of L3/4 and L4/5 in terms of coronal (**I**) and sagittal (**J**) reconstruction

The radiographic outcomes are shown in Table 3. The Cobb angle, PT, PI, LL, SS, and DH were significantly improved immediately after the operation compared with preoperatively, and these improvements were maintained at final follow-up. The mean Cobb angle was 15.0° ± 3.7° preoperatively, 7.2° ± 3.1° immediately postoperatively, and 7.2° ± 3.3° at final follow-up. The mean PT was 25.1° ± 6.9° preoperatively, 18.9° ± 6.0° immediately postoperatively, and 19.2° ± 5.7° at final follow-up. The mean SS was 26.1° ± 8.1° preoperatively, 33.9° ± 8.0° immediately postoperatively, and 34.3° ± 7.4° at final follow-up. The mean LL was 32.2° ± 11.3° preoperatively, 40.3° ± 11.8° immediately postoperatively, and 40.7° ± 11.0° at final follow-up. The mean DH was 0.7 ± 0.3 cm preoperatively, 1.2 ± 0.2 cm immediately postoperatively, and 1.1 ± 0.2 cm at final follow-up. The mean PI was 51.2° ± 8.2° preoperatively, 52.7° ± 7.5° immediately postoperatively, and 53.5° ± 7.6° at final follow-up. At final follow-up, lumbar CT scans indicated that 28 patients (93.33%) had solid interbody fusion. CT images of typical patients are shown in Fig. 3.

**Table 3:** Radiographical results

Before operation		After operation	Last follow-up	*P* value^*^	*P* value^**^
Cobb angle	15.0 ± 3.7	7.2 ± 3.1	7.2 ± 3.3	<0.001	<0.001
PT	25.1 ± 6.9	18.9 ± 6.0	19.2 ± 5.7	<0.001	<0.001
PI	51.2 ± 8.2	52.7 ± 7.5	53.5 ± 7.6	0.45	0.24
SS	26.5 ± 8.1	33.9 ± 8.0	34.3 ± 7.4	<0.001	<0.001
LL	32.2 ± 3.7	40.3 ± 11.8	40.7 ± 11.0	0.007	0.005
DH	0.72 ± 0.27	1.24 ± 0.2	1.14 ± 0.2	<0.001	<0.001

*reflected the difference between before and after operation. **reflected the difference between after operation and last follow-up

## Discussion

Adolescent idiopathic scoliosis (AIS) is the most common type of scoliosis with the prevalence rate of 0.47% - 5.20% in the world at present [18]. Brace therapy has proved to be the most effective conservative treatment for AIS which is applicable to patients with immature bones and lateral bending angle of 20 to 45 degrees [19]. Unlike from AIS, most patients with ADS are older adults who often have reduced bone density and multiple underlying medical comorbidities, and may therefore benefit from a minimally invasive operation. Minimally invasive surgeries such as DLIF/XLIF with or without posterior internal fixation have become widely accepted for the treatment of ADS [9, 14, 20]. However, DLIF/XLIF has a high incidence (10%–20%) of postoperative complications such as lumbar plexus nerve and psoas muscle injuries that lead to thigh pain and gluteal motor weakness, even if intraoperative electrophysiological monitoring is used [21]. In contrast, in OLIF, the intervertebral disc is approached via a corridor between the psoas muscle and abdominal major vessels [22], which may effectively reduce the incidences of complications related to lumbar plexus injury, such as thigh pain and paresthesia. Anatomically, OLIF has a unique advantage in the treatment of ADS because the ideal exposure space for OLIF is L2–L5, which is where ADS mainly occurs. Furthermore, patients with ADS usually have small coronal Cobb angles [23]. For example, a previous study that investigated the safety and efficacy of XLIF with or without supplemented instrumentation in the treatment of ADS reported a mean preoperative Cobb angle of 21.6° [24]. In our study, the mean preoperative Cobb angle was 15.0° (range 11.4° to 23.2°). Compared with conventional posterior surgery, OLIF enables the surgeon to directly manage the intervertebral disc and use larger cage sizes. In the present study cohort, the OLIF cages had a height of 10–14 mm and a length of 45–55 mm. Therefore, the coronal Cobb angle was effectively corrected by the parallel insertion of the OLIF cage with the intervertebral disc distracted. In the present study, the coronal Cobb angle was corrected from a preoperative mean of 15.0° ± 3.7° to a mean of 7.2° ± 3.2° at final follow-up. Furthermore, the LL increased from a preoperative mean of 32.2° ± 11.3° to a mean of 40.7° ± 11.0° at final follow-up. These good results may be attributable to the anterior reconstruction with the OLIF cage. Similar results were reported in a previous study that found a significant increase in segmental lordosis and DH after OLIF [25]. Biomechanical studies have shown that the restoration of LL is beneficial to increase the tension of the anterior longitudinal ligament, which improves the intervertebral fusion rate and reduces the rate of degeneration of adjacent segments [26]. Another study also found that LL restoration is the key to the recovery of the sagittal balance of the lumbar spine [27]. In the present study, the SS increased from 26.1° ± 8.1° preoperatively to 34.3° ± 7.4° at final follow-up. Sagittal imbalance of the lumbar spine is compensated for by a reduction in the SS angle. The forward inclination of the sacrum not only reduces the SS angle, but also reduces the LL angle. The posterior movement of the body balance increases the gravity forces borne by the hip and knee joints, resulting in symptoms such as low back and leg pain. PT decreased from 25.1° ± 6.9° preoperatively to 19.2° ± 5.7° at final follow-up. A high PT tilts the pelvis backward, increases the tension of the lumbosacral ligaments and muscles, and causes lumbosacral pain. Insufficient postoperative improvement of the PT not only prevents the relief of lumbosacral and lower limb pain, but also accelerates the degeneration of adjacent segments. Recovery of the sagittal balance by reducing the PT and increasing the SS reduces the adjacent segmental degeneration caused by the compensatory movements and improves the patient’s symptoms.

It remains controversial whether posterior internal fixation is necessary for OLIF in the treatment of ADS. Some scholars suggest the use of single-stage internal fixation because of concerns that the absence of posterior pedicle screws may cause cage subsidence, which in turn affects clinical outcomes due to the loss of DH [28]. In our opinion, the stand-alone OLIF technique maintains spinal stability without damaging the paraspinal muscles, anterior and posterior longitudinal ligaments, and facet joints compared with traditional posterior approaches such as PLIF and TLIF. In addition, the stand-alone OLIF technique creates a much bigger lateral cage bone-graft window than traditional PLIF and TLIF, which may improve the fusion rate. After the large-sized cage was implanted, it was able to be tightly fixed to the epiphysis ring by the tightened anterior and posterior longitudinal ligaments. Compared with a narrower implant, the placement of a wider cage in the periphery of the endplate results in a lower incidence of implant subsidence owing to its efficacy in providing segmental stability [29]. There are also reports of stand-alone ALIF and stand-alone lateral lumbar interbody fusion achieving good clinical efficacy. Rao et al. [30] reported an overall clinical success rate of 93% in 27 patients diagnosed with low-grade lumbar spondylolisthesis who underwent stand-alone ALIF, while Ahmadian et al. [31] reported that stand-alone lateral interbody fusion achieved good clinical efficacy and outcomes in a cohort of 59 patients, including 18 with ADS. These previous results show that stand-alone minimally invasive lumbar interbody fusion is a viable option for carefully selected patients.

Our data showed that the DH at final follow-up was higher than the preoperative DH. However, the presence of radiographic subsidence is not directly correlated with the clinical outcomes [32]. The VAS and ODI scores were significantly lower at final follow-up compared with preoperatively, which indicated that the present patients achieved a significant improvement in their quality of life. Similarly, Zhang et al. [33] reported that although cage subsidence was found in a total of 15 fused segments among patients who underwent stand-alone OLIF, their symptoms had been alleviated during follow-up.

It is important to note that stand-alone technology has limited indications. We believe that bone density is an important factor that should be considered. Tempel et al. [34] found that the incidence of cage subsidence is significantly higher in patients with bone density T values of − 1.0 to − 2.4 than in those with bone density T values of > − 1.0, and suggested that patients with a bone density T value of < − 1.0 should be treated with posterior pedicle screw fixation. We also do not recommend stand-alone OLIF for patients with osteoporosis (T < 2.5) because the incidence of cage subsidence increases significantly in this population. It is essential to adequately inform patients scheduled for stand-alone OLIF that posterior decompression and internal fixation may be required if their clinical symptoms recur after surgery.

The present study has some limitations. First, the study was retrospective; therefore, although the results confirmed that OLIF achieved good clinical results in patients with ADS, there is still a need for randomized controlled trials of DLIF/XLIF and PLIF. Second, the sample size was small and the follow-up duration was short; therefore, the long-term efficacy and complications require further evaluation. In a future study, we will compare the clinical and radiological outcomes of PLIF versus stand-alone OLIF for the treatment of ADS in a larger sample size with a follow-up of more than 5 years.

We found that stand-alone OLIF in the treatment of ADS achieved significant improvements in the DH, VAS scores, and ODI, with restoration of the sagittal and coronal balance at final follow-up. In our experience, stand-alone OLIF is safe and effective in the treatment of ADS in patients with mild coronal plane deformity caused by indirect decompression and arrested curve progression.

## Supplementary Information

**Additional file 1: Figure S1.** The Cobb angle, sagittal parameters and LL before (A,C and E) and after (B,D and F) operation of case 9.

**Additional file 2: Figure S2.** The Cobb angle, sagittal parameters and LL before (A,C and E) and after (B,D and F) operation of case 23.

**Additional file 3.**

## Data Availability

The datasets used during the current study was available from a supplementary file.
