# Supplementary figures and images for: Clinical and radiographic outcomes of stand-alone oblique lateral interbody fusion in the treatment of adult degenerative scoliosis: a retrospective observational study
Source: BMC Musculoskelet Disord. 2022 Dec 27;23:1133. doi: 10.1186/s12891-022-06035-9 (PMC9793660; doi:10.1186/s12891-022-06035-9)

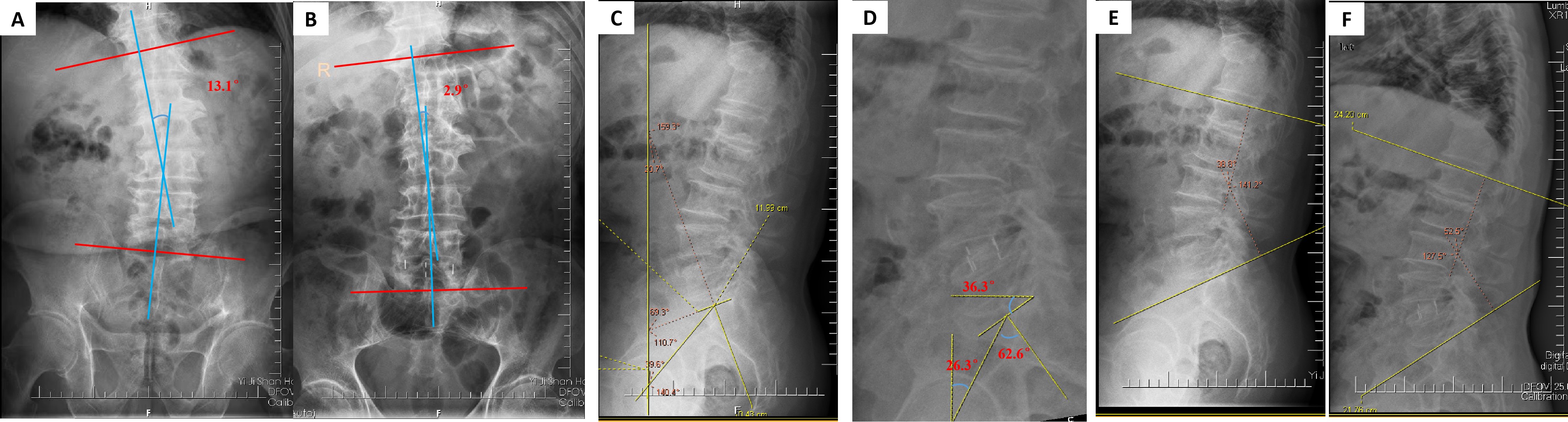

Supplement: Supplementary file 1 — Additional file 1: Figure S1. The Cobb angle, sagittal parameters and LL before (A,C and E) and after (B,D and F) operation of case 9. [file 12891_2022_6035_MOESM1_ESM.jpg]

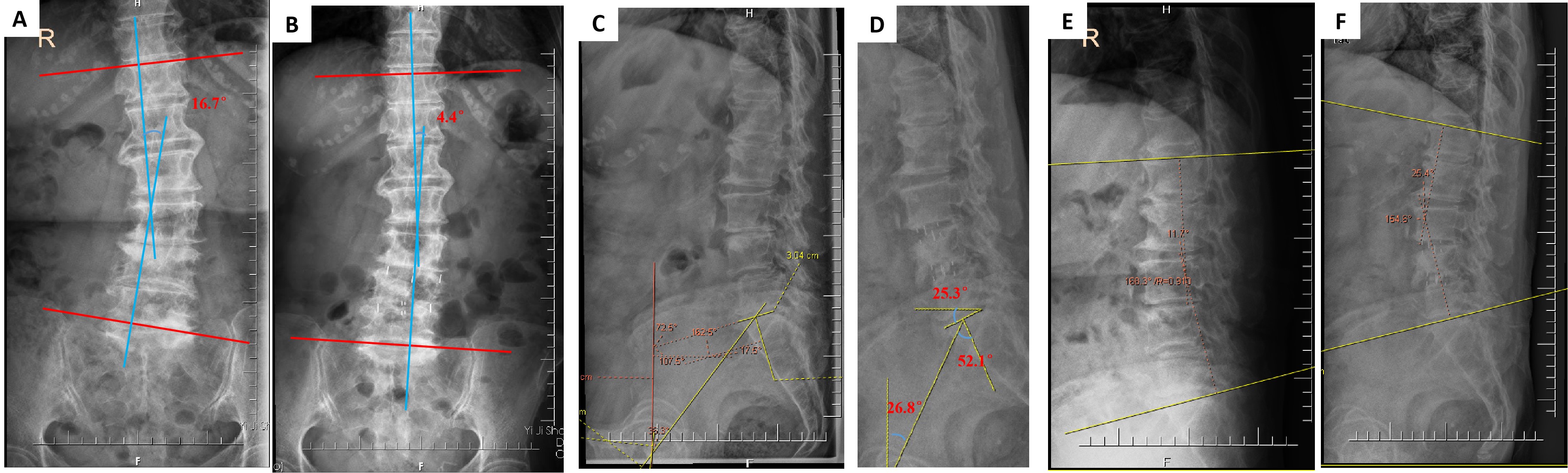

Supplement: Supplementary file 2 — Additional file 2: Figure S2. The Cobb angle, sagittal parameters and LL before (A,C and E) and after (B,D and F) operation of case 23. [file 12891_2022_6035_MOESM2_ESM.jpg]
